# Retraction Note: Production, bioprocessing and antiproliferative activity of camptothecin from *Aspergillus terreus*, endophyte of *Cinnamomum camphora*: restoring their biosynthesis by indigenous microbiome of *C. camphora*

**DOI:** 10.1186/s12934-026-03130-7

**Published:** 2026-09-23

**Authors:** Abeer Eldeghidy, Gamal Abdel-Fattah, Ashraf S. A. El-Sayed, Ghada G. Abdel-Fattah

**Affiliations:** 1 Botany and Microbiology Department, Faculty of Science, Mansoura University, Mansoura, Egypt; 2 Enzymology and Fungal Biotechnology Lab, Botany and Microbiology, Faculty of Science, Zagazig University, Zagazig, Egypt

**Retraction: Microbial Cell Factories (2023) 22:143**

**https://doi.org/10.1186/s12934-023-02158-3**

The Editor-in-Chief has retracted this article. After publication, concerns were raised regarding the data presented in the figures, specifically:

Figure 6A *A. terreus* CPT 0 min and 24 images appear highly similar;

Figure 6A Control 24 h and *A. terreus* CPT 72 h images appear to overlap with different magnification, and also appear highly similar to Fig. 8D in [1], Fig. 7D in [2], and Fig. 4A 72 h 5b/HUVEC in [3];

The gels in Figs. 5A and 9A appear to show signs of splicing.

The Editor-in-Chief therefore no longer has confidence in the presented data.

Ashraf S. A. El-Sayed does not agree with this retraction. Abeer Eldeghidy, Gamal Abdel-Fattah and Ghada G. Abdel-Fattah have not responded to any correspondence from the editor or publisher about this retraction.
